# Diagnostic accuracy and management of suspected acute otitis media: A multi-continental survey of primary care physicians using tympanic membrane images in clinical cases

**DOI:** 10.1080/13814788.2026.2726138

**Published:** 2026-09-11

**Authors:** Ida Lindman, Lisa Ahlstrand, Thorbjörn Lundberg, Anna Moberg, Karin Mossberg, Lidia Caballero, Clare Heal, Samuel Coenen, Ingrid Picard, Hanna Kaduszkiewicz, Catharina Escales, Julia Hansmann-Wiest, Noirin Fitzgerald, Tomoko Ito, Guro Haugen Fossum, Anja Maria Braend, Owen Eales, Sanele Ngcobo, Carl Llor, Oliver Van Hecke, Mark Ebell, Ronny Gunnarsson, Pär-Daniel Sundvall

**Affiliations:** ^a^ Research, Education, Development & Innovation, Primary Health Care, Region Västra Götaland, Gothenburg, Sweden; ^b^ General Practice/Family Medicine, School of Public Health and Community Medicine, Institute of Medicine, Sahlgrenska Academy, University of Gothenburg, Gothenburg, Sweden; ^c^ Family Medicine, Department of Public Health and Clinical Medicine, Umeå University, Umeå, Sweden; ^d^ Department of Health, Medicine and Caring Sciences, Linköping University, Linköping, Sweden; ^e^ San Miguel Primary Health Care Center, Posadas, Public Health Ministry of Misiones, Misiones, Argentina; ^f^ College of Medicine and Dentistry, James Cook University, Queensland, Australia; ^g^ Department of Family Medicine and Population Health (FAMPOP), University of Antwerp, Antwerp, Belgium; ^h^ Vaccine & Infectious Disease Institute (VAXINFECTIO), University of Antwerp, Antwerp, Belgium; ^i^ Institute of General Practice, Kiel University, Kiel, Germany; ^j^ Department of General Practice, School of Medicine, University of Galway, Galway, Ireland; ^k^ Department of Family and Community Medicine, Hamamatsu University School of Medicine, Hamamatsu, Japan; ^l^ General Practice Research Unit, Department of General Practice, University of Oslo, Oslo, Norway; ^m^ Department of Family Medicine, School of Medicine, University of Pretoria, Pretoria, South Africa; ^n^ Institute for Primary Health Care Research Jordi Gol i Gurina (IDIAPJGol), Barcelona, Spain; ^o^ CIBER de Enfermedades Infecciosas, Instituto de Salud Carlos III, Madrid, Spain; ^p^ Centre for General Practice, Department of Public Health and Primary care, Ghent University, Ghent, Belgium; ^q^ Department of Family Medicine, College of Human Medicine, Michigan State University, East Lansing, MI, USA; ^r^ Centre for Antibiotic Resistance Research (CARe) at University of Gothenburg, Gothenburg, Sweden; ^s^ College of Medicine and Dentistry, James Cook University, Cairns, Australia

**Keywords:** Acute otitis media, diagnostic accuracy, primary care, antibiotic prescription

## Abstract

**Background:**

Acute otitis media (AOM) is a leading cause of primary care visits and antibiotic prescriptions in children. Diagnostic uncertainty and variability in management contribute to misdiagnosis and inappropriate antibiotic use.

**Objective:**

This study examines how primary care physicians across countries diagnose and manage suspected AOM through clinical cases.

**Methods:**

International cross-sectional survey using structured clinical cases with validated tympanic membrane images. The survey was administered during professional meetings for primary care physicians in 12 countries (Argentina, Australia, Belgium, Germany, Ireland, Japan, Norway, South Africa, Spain, Sweden, United Kingdom, and the United States).

**Results:**

A total of 1,365 primary care physicians participated. Across all responses to AOM cases, 60% were correctly diagnosed as AOM, while only 31% of responses involving a normal tympanic membrane were correctly identified. Antibiotic prescribing varied substantially across countries and between cases. Multivariable regression analyses showed that diagnostic accuracy was a significant predictor of antibiotic prescribing. Physicians who correctly identified a normal tympanic membrane were significantly less likely to prescribe antibiotics (p < 0.001). Notable differences in prescribing patterns were evident between countries.

**Conclusion:**

Diagnostic accuracy for suspected AOM was generally low among primary care physicians, with wide variation across countries. Antibiotics were frequently prescribed even in the absence of AOM. This underscores the need for improved diagnostic education to reduce unnecessary antibiotic use globally.

## Background

Acute otitis media (AOM) is among the most common infections in children and poses a frequent diagnostic and therapeutic challenge in primary care settings [1,2]. By the age of two, up to 70% of children will have experienced at least one episode of AOM [3]. Due to its high prevalence, AOM places a substantial burden on children, their families, and healthcare systems worldwide [4–6].

The diagnosis is based on clinical symptoms together with clinical findings such as tympanic membrane (TM) inflammation and purulent middle ear effusion [3]. Visualisation of the TM is crucial for identifying signs of AOM, including middle ear effusion. Bulging of the TM is widely considered the most important otoscopic sign of AOM, while opacity, discolouration and reduced mobility provide additional diagnostic support. Otoscopy is the primary diagnostic tool, while pneumatic otoscopy and tympanometry can further support the diagnosis by assessing middle ear effusion [7]. Although these diagnostic tools are widely available in some settings, diagnostic accuracy among primary care physicians remains low [8–10]. Differentiating AOM from other conditions such as otitis media with effusion (OME) or a red TM without effusion, in some countries labelled otitis simplex, is particularly challenging [9].

Furthermore, AOM is a leading cause of antibiotic prescriptions for children [11–13]. Although antibiotic-prescribing rates declined in the early 2000s, this downward trend has since plateaued [13]. Despite efforts to reduce antibiotic use, rising resistance and persistently high prescribing rates in some settings highlight the need for improved diagnostic accuracy and adherence to treatment guidelines [14–16]. Misdiagnosing OME or a red TM without middle ear effusion as AOM increases the risk of inappropriate antibiotic prescribing.

Internationally, multiple guidelines exist for the management of AOM, leading to considerable variability in management [2,9,17–19]. Treatment strategies differ by country and patient age, ranging from immediate antibiotic therapy to a watchful waiting approach [17,20]. Differences in recommendations, particularly in antibiotic use, reflect both regional variations in clinical practice and ongoing uncertainty about optimal management. Additionally, studies evaluating diagnostic accuracy on a global scale are limited, and adherence to national guidelines remains suboptimal [21,22]. Given the consequences of inappropriate antibiotic prescribing, a better understanding of how primary care physicians diagnose and manage AOM is essential.

This study aimed to investigate how primary care physicians in different countries assess patients with suspected AOM using clinical case scenarios accompanied by an image of the TM. Further aims were to assess the rates and appropriateness of antibiotic prescribing, as well as variability in diagnostic and prescribing decisions across countries.

## Methods

This international, cross-sectional, case-based survey was conducted among primary care physicians in 12 countries.

### Subjects

Eligible participants were board-certified general practitioners/family physicians (GPs) and specialist trainees/registrars in general practice/family medicine (STs). Participants worked in publicly or privately run primary health care centres in Argentina, Australia, Belgium, Germany, Ireland, Japan, Norway, South Africa, Spain, Sweden, the United Kingdom, and the United States of America. Physicians were recruited during professional education events unrelated to AOM. These included continuing medical education meetings for GPs, educational days for specialist trainees/registrars and other local or regional primary care meetings where physicians from primary care settings gathered for professional education and exchange. Recruitment at these meetings provided a pragmatic way to recruit physicians from a broad range of primary care settings. Importantly, because these educational events were unrelated to AOM, recruitment was not directed towards physicians with a specific interest or expertise in AOM. All participating physicians reported routinely seeing paediatric patients in clinical practice. Participation was voluntary, and each participant completed a printed version of the questionnaire anonymously.

### Data collection

The questionnaire comprised two sections. The first section collected demographic information, including gender, age, professional status (GP versus ST), and country of practice.

The second section consisted of case-based questions, in which participants were presented with brief fictional patient cases, each accompanied by a validated image of the TM. Participants were asked to diagnose the condition as AOM, OME, a TM with some redness without middle ear effusion (in some countries labelled as otitis simplex), or a normal TM. They were also asked to indicate whether they wished to prescribe antibiotics using standardised multiple-choice options specifying immediate or delayed prescription.

**Figure 1.**
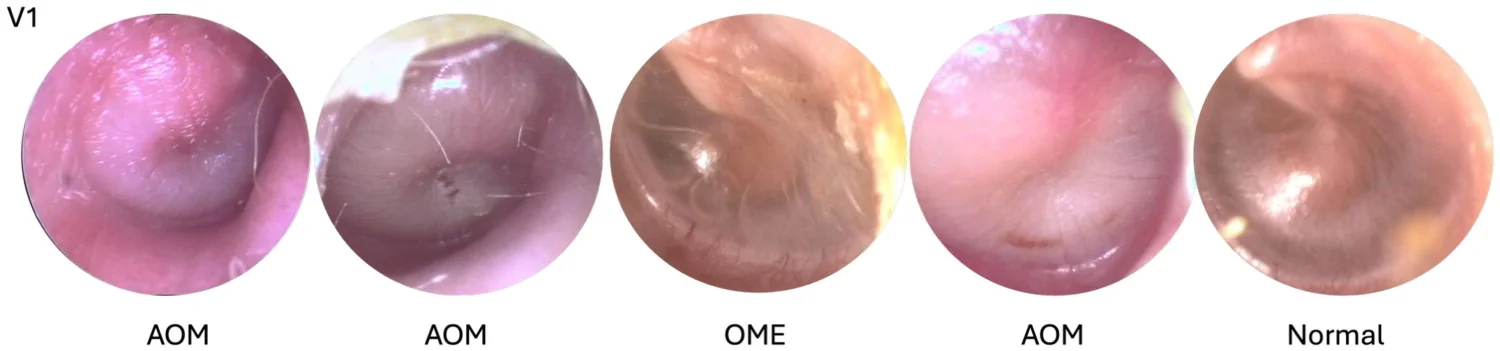
Tympanic membrane images used in Survey version 1. AOM: Acute Otitis Media; OME: Otitis media with effusion.

**Figure 2.**
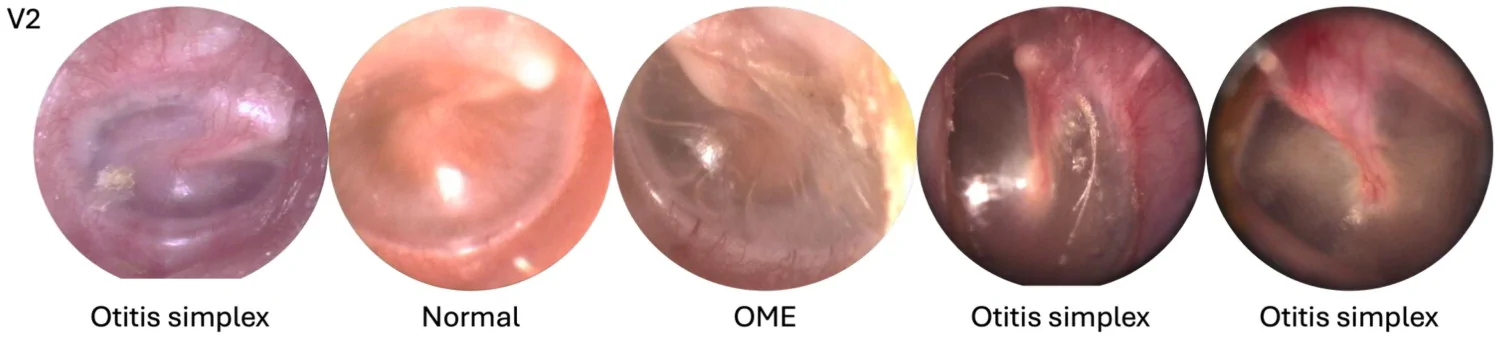
Tympanic membrane images used in Survey version 2.Otitis simplex refer to normal eardrum with some redness. OME: Otitis media with effusion.

To assess whether physicians could distinguish between simple redness of the TM without middle ear effusion and true AOM, two questionnaire versions were distributed (Appendix 1 and 2), each containing five cases. Each case included a brief description accompanied by an image of the TM, including the patient’s age and laterality, as seen in Table 3. All pictures of the TM showed pristine appearances with no wax. To prevent participants from retrospectively adjusting their diagnoses, cases with AOM and TM with some redness were not included in the same version. In one version (Figure 1a and Appendix 1), some cases featured TM images that clearly met the diagnostic criteria for AOM, while in the other version (Figure 2 and Appendix 2), some cases featured TM images consistent with TM with some redness without middle ear effusion. Case descriptions were identical in both versions. One case involved OME and included the same image in both versions. Other case descriptions were paired with different TM images across versions – representing AOM, TM with some redness without middle ear effusion, or a normal TM – to increase variation between cases. The questionnaire versions were randomly distributed in approximately equal numbers at each site to minimise bias.

The TM images used in the questionnaire had been assessed by an expert panel consisting of one otolaryngologist and two board-certified general practitioners/family physicians. The diagnosis assigned to each image was established following a joint review of the TM image, tympanometry findings, clinical history and presented symptoms. The fictional cases were piloted among primary care physicians in participating countries prior to the study to ensure face validity. All TM images used in the questionnaire were anonymised and derived from clinical cases without any identifiable patient information. The accompanying case descriptions were plausible yet fictional and did not represent real patient histories. Therefore, it is not possible to trace any individual from the images used.

### Outcomes

The primary outcomes were physicians’ diagnostic assessments and antibiotic prescribing decisions for each clinical case.

Diagnostic assessment was categorised as AOM, OME, a TM with some redness without middle ear effusion, or a normal TM. Antibiotic prescribing was defined as the physician’s decision to prescribe antibiotics (yes/no). Both immediate and delayed prescriptions were included because the outcome of interest was whether antibiotics were prescribed.

Responses were analysed according to physicians’ actual diagnostic and treatment decisions, regardless of whether these corresponded to the predefined diagnosis established by the expert panel, as the aim was to evaluate physicians’ diagnostic and treatment decision-making.

### Statistics

Descriptive statistics were used for demographic data. Factors associated with antibiotic prescribing were analysed using multivariable binary logistic regression models for each case-based question. The dependent variable was any antibiotic prescription (yes/no). Independent variables were correct diagnosis (as determined by an expert panel), physician’s professional status (ST/GP), age, gender, and country of practice. All analyses were conducted using IBM SPSS Statistics (Version 29.0.2.0). The level of significance was set at p ≤ 0.05.

A priori, a two-tailed sample size calculation was performed with a power of 0.95 and an alpha of 0.05. Assuming that doctors 10 years younger prescribe antibiotics 10% less often, female doctors prescribe antibiotics 20% more often than male doctors, and STs prescribe 15% fewer antibiotics than GPs, the estimated required sample sizes were 345, 326, and 676, respectively [23]. It was therefore determined that at least 676 participants should be included.

### Ethical considerations

The study was conducted in accordance with the Declaration of Helsinki, and ethical approval or exemption was obtained from each country (Appendix 3).

## Results

A total of 1,365 physicians from 12 countries completed the questionnaire between December 2022 and December 2023. The mean age was 39 years (SD 10), 61% were female, and 40% were board-certified general practitioners/family physicians (GPs) (Table 1).

**Table 1.** Demographic characteristics of survey respondents.

Country	Number of respondents*	Mean age in years (standard deviation)	Female (%)	Median year of medical degree (interquartile range)	Being a specialist in general practice** (%)
Argentina	100	36 (11)	57	2019 (2010–2020)	25
Australia	38	43 (14)	76	2002 (1992–2014)	68
Belgium	182	40 (13)	73	2012 (2000–2020)	69
Germany	150	39 (8.4)	73	2015 (2009–2018)	23
Ireland	47	41 (12)	49	2011 (1996–2017)	53
Japan	34	37 (10)	33	2018 (2007–2020)	41
Norway	84	38 (6.9)	76	2015 (2009–2017)	27
South Africa	107	41 (11)	61	2008 (2001–2016)	33
Spain	90	37 (10)	69	2014 (2005–2018)	60
Sweden	358	39 (8.9)	51	2015 (2011–2018)	22
United Kingdom	104	40 (9.4)	59	2011 (2004–2016)	71
USA	44	41 (14)	50	2017 (1998–2020)	61

*Demographic data were available for 1,338 of the 1,365 participants, **Board-certified general practitioner.

### Diagnostic accuracy

Of all responses to AOM cases, 60% (1195/1978) were correctly diagnosed as AOM. For cases of OME, 68% (878/1299) of responses were correct. TM with some redness without middle ear effusion was correctly identified in 28% (547/1930) of responses, and a normal TM in 31% (402/1296). There were substantial differences in diagnostic accuracy between countries. Sweden and Spain had the highest accuracy rates for AOM diagnoses (85% (473/559) and 80% (122/152), respectively), whereas Germany had the lowest rate of correct AOM diagnoses (29% (66/229)). Regarding correct assessment of normal TMs, Spain had the highest rate (52% (46/89)), followed by the UK (45% (47/104)), while Australia had no correct assessments of normal TMs (0/39) (Table 2).

**Table 2.** Diagnostic accuracy by country.

	Correct diagnosis^a^ % (n/N)^b^			
Country	Acute otitis media	Otitis media with effusion	Otitis simplex^c^	Normal eardrum
Argentina	49 (78/158)	69 (69/100)	21 (30/140)	24 (24/100)
Australia^d^	---	79 (31/39)	34 (38/112)	0.0 (0/39)
Belgium	52 (151/289)	48 (80/165)	27 (57/213)	37 (60/164)
Germany	29 (66/229)	55 (83/151)	26 (61/231)	33 (50/153)
Ireland	42 (42/99)	67 (32/48)	5.3 (2/38)	40 (18/45)
Japan	75 (9/12)	62 (26/42)	24 (27/113)	7.0 (3/43)
Norway	57 (75/131)	59 (47/79)	19 (22/113)	35 (28/80)
South Africa	45 (58/130)	68 (54/80)	31 (38/123)	23 (19/82)
Spain	80 (122/152)	78 (70/90)	19 (22/116)	52 (46/89)
Sweden	85 (473/559)	81 (288/357)	35 (179/506)	27 (95/352)
United Kingdom	56 (85/153)	66 (69/104)	34 (53/156)	45 (47/104)
USA	55 (36/66)	66 (29/44)	26 (18/69)	27 (12/45)
All countries	60 (1195/1978)	68 (878/1299)	28 (547/1930)	31 (402/1296)

^a^Gold standard refers to the diagnosis established by the expert group (see Methods section).

^b^Each survey included multiple clinical cases, so the number of responses exceeds the number of returned surveys. Percentages reflect the proportion of correct responses across all relevant cases, not the number of respondents who correctly diagnosed all cases within a category.

^c^Normal eardrum with some redness; in some countries labelled otitis simplex. Does not warrant antibiotic treatment.

^d^In Australia, only the survey version without acute otitis media (AOM) cases was distributed.

### Antibiotic prescription patterns

Antibiotic prescription rates varied significantly between countries and across different cases and ranged from 17% to 82% depending on the clinical case, with consistently higher prescribing for classical presentations of AOM. Countries such as Argentina and South Africa generally demonstrated higher prescribing rates, whereas Belgium and Sweden showed more restrictive prescribing patterns (Table 3).

**Table 3.** Proportion of antibiotic prescriptions across all clinical cases.

	Acute otitis media (AOM)			Effusion	Otitis simplex^a^			Normal eardrum	
Age:	4 years	8 months	9 years	13 years	4 years	9 years	18 months	8 months	18 months
Bilateral:	Yes	No	No	No	Yes	No	Yes	No	---
Temperature:	38.2 °C 100.8 °F	38.3 °C 100.9 °F	38.0 °C 100.4 °F	Unknown	38.2 °C 100.8 °F	38.0 °C 100.4 °F	38.0 °C 100.4 °F	38.3 °C 100.9 °F	38.0 °C 100.4 °F
Early recurrence of AOM:	No	No	Yes	NA	NA	NA	NA	NA	NA
Antibiotic prescription % (n/N)^b^									
Argentina	42 (22/53)	89 (47/53)	58 (31/53)	71 (71/100)	34 (16/47)	77 (36/47)	64 (30/47)	85 (40/47)	40 (21/53)
Australia^c^	---	---	---	18 (7/39)	42 (16/38)	34 (13/38)	18 (7/39)	59 (23/39)	---
Belgium	11 (11/99)	58 (56/96)	33 (31/94)	4.2 (7/165)	5.3 (4/75)	29 (20/70)	41 (28/68)	28 (20/72)	5.4 (5/92)
Germany	36 (27/76)	75 (58/77)	51 (39/77)	23 (35/152)	28 (22/78)	67 (52/78)	57 (44/77)	51 (39/77)	23 (18/77)
Ireland	44 (15/34)	89 (31/35)	57 (20/35)	47 (22/47)	7.7 (1/13)	62 (8/13)	62 (8/13)	69 (9/13)	27 (9/33)
Japan	25 (1/4)	100 (4/4)	25 (1/4)	63 (25/40)	31 (11/35)	86 (30/35)	63 (22/35)	72 (28/39)	50 (2/4)
Norway	31 (13/42)	84 (37/44)	77 (34/44)	20 (16/82)	15 (6/41)	72 (28/39)	61 (23/38)	58 (23/40)	13 (5/39)
South Africa	67 (31/46)	84 (36/43)	66 (27/41)	49 (39/80)	59 (27/46)	59 (23/39)	63 (24/38)	79 (33/42)	30 (12/40)
Spain	51 (26/51)	96 (49/51)	84 (42/50)	14 (13/90)	31 (12/39)	64 (25/39)	51 (20/39)	54 (21/39)	14 (7/51)
Sweden	32 (61/190)	93 (176/189)	43 (80/188)	7.8 (28/359)	11 (18/171)	31 (52/166)	36 (60/167)	66 (112/170)	11 (21/188)
United Kingdom	54 (28/52)	61 (31/51)	67 (35/52)	19 (19/100)	35 (18/52)	69 (36/52)	50 (26/52)	67 (35/52)	9.6 (5/52)
USA	38 (8/21)	82 (18/22)	73 (16/22)	23 (10/44)	26 (6/23)	61 (14/23)	48 (11/23)	61 (14/23)	32 (7/22)
All countries	36 (243/668)	82 (543/665)	54 (356/660)	22 (292/1298)	24 (157/658)	53 (337/639)	48 (303/636)	61(397/653)	17 (112/651)

NA: Not applicable.

^a^Normal eardrum with some redness; in some countries labelled otitis simplex. Does not warrant antibiotic treatment.

^b^Each survey included multiple clinical cases, so the number of responses exceeds the number of returned surveys. Values are presented as % (n/N), where N represents the total number of respondents from each country answering the specific case. Percentages reflect the proportion of responses in which antibiotics were prescribed across all relevant cases, rather than the proportion of respondents prescribing antibiotics in all cases within a category.

^c^In Australia, only the survey version without acute otitis media (AOM) cases was distributed.

Because few surveys had missing data, most responses could be included in the multivariable regression analyses (Appendix 4, Supplementary Table 1). Correct diagnoses of AOM or OME were independently associated with higher odds of antibiotic prescription, whereas correct identification of otitis simplex or a normal TM was consistently associated with lower odds of antibiotic prescribing. Country-level differences remained after adjustment for physician and case-related characteristics, with several countries showing higher odds of antibiotic prescribing than Sweden (reference), whereas Belgium showed lower odds in several cases. Physician age and gender were not associated with antibiotic prescribing. Board-certified GPs were less likely than STs to prescribe antibiotics in OME cases.

## Discussion

This large, multicentre international study, including 12 countries across six continents, highlights notable discrepancies in both the accuracy of TM assessments and antibiotic prescribing.

Diagnostic accuracy varied considerably between countries. Many clinicians misclassified non-AOM as AOM. The cases represented clear examples of AOM and non-AOM based on tympanic membrane appearance, making differences in diagnostic accuracy unlikely to reflect interpretation of borderline criteria. The findings are consistent with previous research showing that accurate interpretation of TM findings is challenging for physicians [10,11,24]. One previous study reported that 50% of AOM diagnoses were not supported by physical examination findings [25]. This underscores that AOM cannot be diagnosed based on patient history alone, as accurate diagnosis relies primarily on visualisation of the TM [26]. Although clinical conditions may limit otoscopic visibility, this study provided high-resolution, magnified TM images, and the observed variation highlights the need to strengthen training in TM assessment.

Antibiotic prescribing showed substantial variation across both countries and case scenarios (Table 3). For example, antibiotics were prescribed in 36% of responses to a case involving bilateral AOM in a 4-year-old child, with national rates ranging from 11% to 67%. In contrast, for an 8-month-old child with AOM, the overall prescribing rate was 82%, ranging from 58% to 100%. Notably, antibiotic prescribing also varied substantially for conditions where antibiotics provide no benefit, including OME and children with a normal TM. In one scenario involving an 8-month-old child with a normal TM, 61% of respondents prescribed antibiotics, with country-specific rates ranging from 28% (in Belgium) to 85%. In contrast, a case involving an 18-month-old with a normal TM resulted in antibiotic prescriptions in only 17% of responses. This type of variation has previously been observed in a study comparing annual antibiotic prescribing for AOM across countries [16], although that study was limited to five European countries.

While clinical guidelines differ internationally, most agree that AOM often resolves without antibiotics [19,20,27]. Notably, guidelines in both high- and low-income countries share more similarities than differences and generally recommend more restrictive antibiotic use [17]. Antibiotics are typically recommended only in specific clinical scenarios, while cases involving OME, a TM with some redness without middle ear effusion, or a normal TM are not considered indications for antibiotics [28]. Previous studies suggest that educational interventions targeting physicians can improve prescribing practices [29]. The observed diagnostic and prescribing variability suggests that antimicrobial stewardship should prioritise accurate diagnosis of tympanic membrane findings as the foundation for appropriate antibiotic use. Educational interventions should emphasise recognition of a distinctly bulging TM as a key diagnostic feature, differentiation from OME and a normal TM, and adherence to recommendations on delayed rather than immediate antibiotic prescribing when appropriate. Importantly, a diagnosis of AOM should not be based on non-specific symptoms alone.

The substantial variation in both diagnostic and antibiotic prescription practices is noteworthy, even between countries with relatively similar healthcare systems. For instance, Germany and Belgium, despite having comparable structures, demonstrated significant differences. Prior literature has described similar patterns, where some countries adopt more liberal prescribing behaviour regardless of clinical indications [17,22]. Given that therapeutic needs should not differ across high-income settings, this variation is likely due to differences in prescribing culture, levels of guideline adherence, or patient expectations.

In some countries, children with AOM are primarily managed by paediatricians rather than GPs. Consequently, participating GPs may have had less clinical experience with AOM, which could have partly contributed to the observed variability in diagnosis and management across countries. In addition, countries such as South Africa and Argentina may have higher antibiotic use due to contextual factors, including lower vaccination coverage and limited follow-up capacity, which previous research suggests contribute to more liberal prescribing [30]. However, such factors do not explain the observed tendency to prescribe antibiotics in cases without AOM. This pattern calls for further investigation into drivers of overprescribing, including prescribing norms, patient expectations, healthcare accessibility, and system constraints.

## Strengths and limitations

A major strength of this study is its multinational design, encompassing 12 countries across six continents. The large sample size, prospective design, clinically validated images, randomisation of surveys, and case-based methodology provide valuable insights into global diagnostic and treatment practices. Including clinical histories was a further strength, as the objective was to assess routine clinical decision-making rather than image interpretation alone.

Limitations must be acknowledged. In Australia, only the questionnaire without AOM cases was distributed due to practical constraints. Although laterality was indicated, only one eardrum was presented. The term ‘otitis simplex’, defined in the questionnaire as ‘normal eardrum with some redness’a, is not universally used and may have been interpreted as a normal TM in some settings. Clinically, this distinction is minor, as neither condition warrants antibiotic treatment.

Another limitation is the inability to compare treatment choices with national guidelines. It was assumed that diagnostic criteria for AOM would be largely similar across countries, given the broadly consistent clinical definitions in international literature. While most participating countries have national guidelines for AOM, these were not available in all settings. Moreover, guidelines are often not sufficiently detailed to determine the appropriate management for all scenarios in the survey. In particular, the case involving recurrence that did not meet the formal criteria for recurrent AOM illustrated the limitations of current guidelines where most lack clear recommendations regarding antibiotics in such scenarios. Given these limitations, it was decided not to evaluate the alignment of individual treatment decisions with national guidelines. Instead, each case presented in Tables 3 and Appendix 4, Supplementary Table 1 is accompanied by a concise summary of key clinical parameters, allowing readers to interpret the scenarios in light of their own clinical guidelines and contextual practices, and to compare observed antibiotic prescribing patterns across countries accordingly. Furthermore, complete information regarding the number of distributed versus collected questionnaires was not available for all countries. Recruitment at continuing medical education and professional meetings may have resulted in selection bias, as participating physicians may be more engaged in professional development than primary care physicians generally. Thus, diagnostic performance may represent an overestimate of routine practice. Finally, in some countries, paediatricians primarily provide paediatric care, although shortages of specialists mean that GPs and trainees in general practice/family medicine increasingly provide care for children. All participants in this study were recruited from general practice/family medicine settings.

## Conclusion

This study demonstrated that the accuracy of tympanic membrane assessments was generally low, with substantial variation between countries. Likewise, antibiotic prescribing varied markedly, with antibiotics prescribed for both AOM and conditions that do not warrant antibiotic treatment, including OME, otitis simplex, and normal tympanic membranes. These findings underscore the need to strengthen diagnostic accuracy and promote more consistent, evidence-based use of antibiotics in primary care.

## Supplementary Material

Supplemental Material

Supplemental Material

Supplemental Material

Supplemental Material

## Data Availability

Data will be available upon request.
