# Supplementary material for: Diagnostic accuracy and management of suspected acute otitis media: A multi-continental survey of primary care physicians using tympanic membrane images in clinical cases
Source: Eur J Gen Pract. 2026 Sep 11;32(1):2726138. doi: 10.1080/13814788.2026.2726138 (PMC13573547; doi:10.1080/13814788.2026.2726138)
Supplement: Supplementary file 1 [file IGEN_A_2726138_SM2666.docx]

**INFORMATION SHEET**

PROJECT TITLE: ***Perception of best management of suspected acute otitis media in relation to national guidelines – an international study in primary health care***

You are invited to participate in a research project aiming to investigate your perception of best management of suspected acute otitis media. It involves answering a seven-page survey on your approach to patients with a suspected acute otitis media.

Uncertainty when diagnosing a condition can potentially result in either over- or underdiagnosis with entailed risks. We do not presently know how primary care physicians assess pictures of the tympanic membrane.

The aim of this study is to investigate how physicians working in primary health care manage patients with suspected acute otitis media, and furthermore, to investigate the relationship between the same physicians’ perception of how to treat those conditions in relation to current clinical guidelines in each country.

This survey on acute otitis media includes five cases with images of tympanic membranes. You will be asked to associate each case with any of the following conditions: acute otitis media, otitis media with effusion, otitis simplex (normal eardrum with some redness) or a completely normal pale tympanic membrane. Each case will have follow-up questions about your subsequent actions. At the end of the survey, you will be asked to grade the importance of various aspects of the history and physical examination.

Participation is completely voluntary. Your responses will be strictly anonymous, and your response can’t be linked to you. The data from the study will be used in research publications and reports. You will not be identified in any way in these publications.

By studying how physicians in primary health care assess cases of suspected acute otitis media we can investigate the potential for improvement in diagnostic accuracy. The project will also increase knowledge about how current guidelines for acute otitis media have been implemented in primary care in different countries. The results will form the basis for developing nationally adapted antimicrobial stewardship programs for acute otitis media.

This study has been approved by the XXX human research Ethical Review Authority and given the registration number XXX.

If you have any questions about the study, please contact:

**Questionnaire**

Year of medical degree: _______	Age:_______ Gender: ☐Male ☐ Female
Your title: ☐ General Practitioner ☐ Specialist trainee/registrar in primary care
 ☐ Other _________

1. Four-year-old Hermione has had a cold for the last 3 days with symptoms such as coughing and a runny nose. Yesterday, she got bilateral ear pain and fever. She did not sleep well and the pain in her ears continued this morning. You find that she has a body temperature of 100.8°F (38.2°C). Her mother has given her paracetamol which significantly reduced her ear pain, and she is currently happily playing with a toy. When you examine Hermione, both her ears look like this:

Diagnosis? ☐ Acute Otitis Media ☐ Otitis Media with effusion
☐ Otitis simplex (normal eardrum with some redness) ☐ Normal eardrum

What do you do next?

☐ Antibiotics 5 days ☐ Antibiotics 7 days ☐ Antibiotics 10 days
 If antibiotics: ☐ for immediate use ☐ delayed prescription (to take if no improvement)

☐ No antibiotics, symptomatic treatment, and advice to return if not well or worse

2. Hilary turns 8 months next week, and today she visits your practice together with her father. She has been whiney for the last two days, not been eating normally and has slept poorly. Last night her temperature reached 103.1°F (39.5°C) but this morning it was down to 100.9°F (38.3°C). Hilary dislikes the physical examination, but you manage to get a glimpse of her eardrums. The left eardrum is normal, and her right eardrum looks like this:

Diagnosis? ☐ Acute Otitis Media ☐ Otitis Media with effusion
☐ Otitis simplex (normal eardrum with some redness) ☐ Normal eardrum

What do you do next?

☐ Antibiotics 5 days ☐ Antibiotics 7 days ☐ Antibiotics 10 days
 If antibiotics: ☐ for immediate use ☐ delayed prescription (to take if no improvement)

☐ No antibiotics, symptomatic treatment, and advice to return if not well or worse

3. Thirteen-year-old Harry had an acute otitis media in his left ear 3 weeks ago which was successfully treated with antibiotics. The last few days he’s been feeling unwell, and both the cough and the cold is back. He feels as if he has a fever, but they haven’t taken his temperature. He perceives his hearing as impaired. His right eardrum is normal. This is a picture of his left eardrum:

Diagnosis? ☐ Acute Otitis Media ☐ Otitis Media with effusion
☐ Otitis simplex (normal eardrum with some redness) ☐ Normal eardrum

What do you do next?

☐ Antibiotics 5 days ☐ Antibiotics 7 days ☐ Antibiotics 10 days
 If antibiotics: ☐ for immediate use ☐ delayed prescription (to take if no improvement)

☐ No antibiotics, symptomatic treatment, and advice to return if not well or worse

4. Nine-year-old Daniel had an acute otitis media in his left ear 2 weeks ago. Yesterday his left ear started to hurt again. Paracetamol provides partial pain relief and he now has a body temperature of 100.4°F (38.0°C). The right eardrum is normal, and the left looks like this:

Diagnosis? ☐ Acute Otitis Media ☐ Otitis Media with effusion
☐ Otitis simplex (normal eardrum with some redness) ☐ Normal eardrum

What do you do next?

☐ Antibiotics 5 days ☐ Antibiotics 7 days ☐ Antibiotics 10 days
 If antibiotics: ☐ for immediate use ☐ delayed prescription (to take if no improvement)

☐ No antibiotics, symptomatic treatment, and advice to return if not well or worse

5. Eighteen-month-old Elisabeth has had frequent colds for the last six months. Yesterday her mother noticed that Elisabeth reached for her ears. Elisabeth looks a bit tired when you see her sitting in her mother’s lap. She has a body temperature of 100.4°F (38.0°C). Both eardrums look similar and the left one looks like this:

Diagnosis? ☐ Acute Otitis Media ☐ Otitis Media with effusion
☐ Otitis simplex (normal eardrum with some redness) ☐ Normal eardrum

What do you do next?

☐ Antibiotics 5 days ☐ Antibiotics 7 days ☐ Antibiotics 10 days
 If antibiotics: ☐ for immediate use ☐ delayed prescription (to take if no improvement)

☐ No antibiotics, symptomatic treatment, and advice to return if not well or worse

6. How important do you consider the following factors to be in the management of patients with suspected acute otitis media?

|  | **Irrelevant** | **Not that important** | **Somewhat important** | **Very important** | **Not available** |
| --- | --- | --- | --- | --- | --- |
| **History** |  |  |  |  |  |
| Duration of symptoms | ☐ | ☐ | ☐ | ☐ |  |
| Intensity of pain | ☐ | ☐ | ☐ | ☐ |  |
| Patient´s age | ☐ | ☐ | ☐ | ☐ |  |
| History of recurrent AOM | ☐ | ☐ | ☐ | ☐ |  |
| Known impaired sensorineural hearing | ☐ | ☐ | ☐ | ☐ |  |
| Significant impaired immunity | ☐ | ☐ | ☐ | ☐ |  |
| Patient´s/parents requesting antibiotics | ☐ | ☐ | ☐ | ☐ |  |
| **Physical examination** |  |  |  |  |  |
| Patient´s general appearance | ☐ | ☐ | ☐ | ☐ |  |
| Bilateral ear drum pathology | ☐ | ☐ | ☐ | ☐ |  |
| Secretion from  the auditory canal | ☐ | ☐ | ☐ | ☐ |  |
| Tympanic membrane is translucent or opaque | ☐ | ☐ | ☐ | ☐ |  |
| Tympanic membrane redness | ☐ | ☐ | ☐ | ☐ |  |
| Tympanic membrane bulging | ☐ | ☐ | ☐ | ☐ |  |
| Tympanic membrane mobility | ☐ | ☐ | ☐ | ☐ | ☐ |
| **Laboratory tests** |  |  |  |  |  |
| Body temperature | ☐ | ☐ | ☐ | ☐ | ☐ |
| Outcome of  white blood cell count (WBC) and/or  C-reactive protein (CRP) | ☐ | ☐ | ☐ | ☐ | ☐ |
| The outcome of tympanometry | ☐ | ☐ | ☐ | ☐ | ☐ |

**Thank you for participating**
