# Supplementary material for: Diagnostic accuracy and management of suspected acute otitis media: A multi-continental survey of primary care physicians using tympanic membrane images in clinical cases
Source: Eur J Gen Pract. 2026 Sep 11;32(1):2726138. doi: 10.1080/13814788.2026.2726138 (PMC13573547; doi:10.1080/13814788.2026.2726138)
Supplement: Supplementary file 3 [file IGEN_A_2726138_SM2894.docx]

Appendix 3:

### Ethical considerations

The study was conducted in accordance with the Declaration of Helsinki, and ethical approval or exemption was obtained from each participating country as following:

- Argentina: Approved by the Provincial Research Ethics Committee (CEIP) of the Ministry of Health of the Province of Misiones (3 March 2023).
- Australia: Approved by JCU Human Research Ethics Committee (H9126).
- Belgium: Approved by the Ethics Committee of the Antwerp University Hospital and the University of Antwerp (Project ID 5458).
- Germany: Approved by the Ethics Committee of the Medical Faculty, Christian-Albrechts-University, Kiel (D 471/23).
- Ireland: Approved by the ICGP Research Ethics Committee (ICGP_REC_22_021; 15 November 2022).
- Japan: Approved by Toyota Regional Medical Center (2022-KENRIN13).
- Norway: Ethical approval was not required, as the study did not involve patient data.
- South Africa: Approved by the Research Ethics Committee of the Faculty of Health Sciences, University of Pretoria (753/2022).
- Spain: Approved by the Primary Care Research Institute Jordi Gol Ethical Committee Board, Barcelona (23/019).
- Sweden: The Swedish Ethical Review Authority determined that no formal approval was required, as no sensitive personal data were collected (Dnr 2022-05483-01).
- United Kingdom: Approved by the University of Oxford Research Ethics Committee (R85864/RE001; 5 May 2023).
- United States of America: The University of Georgia (UGA) Institutional Review Board reviewed the study and determined that it did not involve research on human subjects as defined by DHHS and FDA regulations; therefore, formal approval was not required (Project 00006012).
