# Supplementary material for: Diagnostic accuracy and management of suspected acute otitis media: A multi-continental survey of primary care physicians using tympanic membrane images in clinical cases
Source: Eur J Gen Pract. 2026 Sep 11;32(1):2726138. doi: 10.1080/13814788.2026.2726138 (PMC13573547; doi:10.1080/13814788.2026.2726138)
Supplement: Supplementary file 4 [file IGEN_A_2726138_SM2908.docx]

Appendix 4– mentioned Supplementary Table 1.

S.**Table 1 – Factors associated with antibiotic prescribing presented with odds ratio and 95% confidence intervals**

|  | Acute otitis media (AOM) | | | Effusion | Otitis simplex | | | Normal eardrum | |
| --- | --- | --- | --- | --- | --- | --- | --- | --- | --- |
| Age: | 4 years | 8 months | 9 years | 13 years | 4 years | 9 years | 18 months | 8 months | 18 months |
| Bilateral: | Yes | No | No | No | Yes | No | Yes | No | --- |
| Fever: | 38.2°C | 38.3° | 38.0°C | Unknown | 38.2°C | 38.0°C | 38.0°C | 38.3°C | 38.0°C |
| Early recurrence of AOM: | No | No | Yes | --- | --- | --- | --- | --- | --- |
|  |  |  |  |  |  |  |  |  |  |
|  |  |  |  |  |  |  |  |  |  |
| Number of questionnaire responses included in regression | 634 | 634 | 625 | 1231 | 622 | 605 | 597 | 616 | 615 |
| Correct diagnosis | 4.9 (3.1-7.6)  p=<0.001 | 3.9 (2.4-6.4)  p=<0.001 | 10.4 (6.6-16.5)  p=<0.001 | 3.2 (2.2-4.7)  p=<0.001 | 0.081 (0.038-0.17)  p<0.001 | 0.047 (0.025-0.089)  p<0.001 | 0.060 (0.033-0.12)  p<0.001 | 0.0080 (0.0020-0.032)  p<0.001 | 0.0050 (0.0010-0.040)  p<0.001 |
| Being a GP | 0.96 (0.61-1.5)  p=0.86 | 0.84 (0.50-1.4)  p=0.53 | 1.1 (0.70-1.7)  p=0.71 | 0.45 (0.30-0.66)  p=<0.001 | 0.90 (0.53-1.5)  p=0.69 | 0.73 (0.46-1.2)  p=0.19 | 1.2 (0.78-1.9)  p=0.40 | 0.66 (0.42-1.0)  p=0.074 | 0.85 (0.47-1.6)  p=0.61 |
| Physician age (decades) | 1.0 (0.85-1.3)   p=0.75 | 1.2 (0.96-1.5)  p=0.10 | 1.0 (0.82-1.2)  p=0.98 | 0.92 (0.77-1.1)  p=0.39 | 1.2 (0.92-1.5)  p=0.20 | 1.0 (0.83-1.3)  0.80 | 0.83 (0.68-1.0)  p=0.080 | 1.0 (0.83-1.3)  p=0.81 | 1.0 (0.76-1.3)  p=0.99 |
| Physician being female | 1.1 (0.77-1.6)  p=0.54 | 1.4 (0.84-2.2)  p=0.22 | 1.5 (1.0-2.3)  p=0.038 | 0.94 (0.67-1.3)  p=0.73 | 1.4 (0.86-2.2)  p=0.19 | 1.4(0.92-2.1)  p=0.12 | 1.3 (0.88-2.0)  p=0.17 | 1.6 (1.0-2.4)  p=0.042 | 1.3 (0.79-2.3)  p=0.28 |
| Country |  |  |  |  |  |  |  |  |  |
| Argentina | 2.9 (1.4-5.9)  p=0.003 | 1.1 (0.38-3.3)  p=0.84 | 4.9 (2.2-11)  p=<0.001 | 45 (24-85)  p<0.001 | 6.5 (2.7-16)  p<0.001 | 6.0(2.6-14)  p<0.001 | 2.2 (1.0-4.8)  p=0.044 | 2.7 (1.0-7.3)  p=0.049 | 9.0 (3.7-22)  p<0.001 |
| Australia** | --- | --- | --- | 4.9 (1.9-13)  p=0.001 | 4.3 (1.7-11)  p=0.002 | 1.6 (0.63-4.2)  p=0.31 | 0.26 (0.086-0.77)  p=0.16 | 0.46 (0.21-1.0)  p=0.55 | --- |
| Belgium | 0.42 (0.19-0.91)  p=0.027 | 0.20 (0.088-0.44)  p=<0.001 | 0.68 (0.36-1.3)  p=0.24 | 1.1 (0.47-2.8)  p=0.78 | 0.56 (0.17-1.8)  p=0.33 | 0.82 (0.41-1.6)  0.57 | 0.69 (0.35-1.3)  p=0.26 | 0.16 (0.079-0.31)  p<0.001 | 0.65 (0.21-2.0)  p=0.45 |
| Germany | 3.7 (1.9-7.6)  p=<0.001 | 0.57 (0.24-1.3)  p=0.20 | 3.9 (2.0-7.8)  p=<0.001 | 5.7 (3.2-10.2)  p<0.001 | 3.9 (1.8-8.6)  p<0.001 | 3.8(1.9-7.3)  p<0.001 | 2.4 (1.2-4.7)  p=0.013 | 0.47 (0.24-0.91)  p=0.026 | 3.1 (1.4-6.9)  p=0.007 |
| Ireland | 3.1 (1.2-7.9)  p=0.017 | 1.5 (0.38-6.1)  p=0.55 | 3.4 (1.3-9.2)  p=0.014 | 20 (8.8-43.5)  p<0.001 | 0.66 (0.078-5.6)  p=0.70 | 1.8(0.53-6.0)  p=0.35 | 2.1 (0.54-7.8)  p=0.29 | 0.77 (0.19-3.1)  p=0.71 | 6.4 (2.0-21)  p=0.002 |
| Japan | 0.59 (0.060-5.9)  p=0.65 | --- | 0.31 (0.031-3.1)  p=0.32 | 38 (16-94)  p<0.001 | 4.5 (1.6-13)  p=0.005 | 12(3.4-39)  p<0.001 | 2.6 (0.93-7.3)  p=0.068 | 1.2 (0.45-3.3)  p=0.70 | 5.0(0.65-38)  p=0.12 |
| Norway | 1.7(0.77-3.8)  p=0.19 | 0.58 (0.21-1.6)  p=0.30 | 9.7 (3.9-24)  p=<0.001 | 3.3 (1.6-7.1)  p=0.001 | 1.2 (0.40-3.6)  p=0.75 | 5.8 (2.3-14)  p<0.001 | 1.9 (0.80-4.6)  p=0.15 | 0.82 (0.34-2.0)  p=0.66 | 1.5 (0.49-4.8)  p=0.47 |
| South Africa | 9.9 (4.5-22)  p=<0.001 | 0.81 (0.28-2.3)  p=0.69 | 7.6 (3.2-18)  p=<0.001 | 19 (9.9-36)  p<0.001 | 16 (6.4-38)  p<0.001 | 4.7 (2.0-11)  p<0.001 | 5.4 (2.1-14)  p<0.001 | 1.9 (0.74-4.9)  p=0.18 | 4.4 (1.7-11)  p=0.002 |
| Spain | 3.1 (1.5-6.3)  p=0.002 | 2.1 (0.44-9.7)  p=0.36 | 9.0 (3.5-23)  p<0.001 | 3.1 (1.5-6.5)  p=0.003 | 2.9 (1.2-7.1)  p=0.022 | 8.6 (3.3-23)  p<0.001 | 1.1 (0.51-2.5)  p=0.76 | 1.7 (0.60-5.0)  p=0.32 | 2.9 (0.96-8.6)  p=0.058 |
| Sweden | (reference) | (reference) | (reference) | (reference) | (reference) | (reference) | (reference) | (reference) | (reference) |
| United Kingdom | 4.4 (2.1-9.6)  p=<0.001 | 0.21 (0.084-0.53)  p=<0.001 | 3.9 (1.8-8.9)  p<0.001 | 5.4 (2.7-11)  p<0.001 | 4.8 (2.1-11)  p<0.001 | 11(4.5-26)  p<0.001 | 3.6 (1.6-8.5)  p=0.003 | 1.7 (0.72-4.2)  p=0.22 | 2.3 (0.67-7.8)  p=0.19 |
| USA | 1.9 (0.56-6.3)  p=0.30 | 0.44 (0.12-1.7)  p=0.22 | 5.0 (1.6-20)  p=0.009 | 8.1 (3.4-20)  p<0.001 | 3.7 (1.2-12)  p=0.028 | 7.2(2.3-23)  p<0.001 | 1.1 (0.41-3.0)  p=0.84 | 0.80 (0.29-2.2)  p=0.66 | 6.4 (1.6-25)  p=0.008 |
